# The determinants of Italian NEETs and the effects of the economic crisis

**DOI:** 10.1186/s41118-018-0031-0

**Published:** 2018-03-22

**Authors:** Claudio Quintano, Paolo Mazzocchi, Antonella Rocca

**Affiliations:** Department of Management and Quantitative Studies, University of Naples “Parthenope”, Via G. Parisi 13, 80132 Naples, Italy

**Keywords:** NEETs, Inactivity, Social exclusion, J13, J24

## Abstract

In recent years, the share of young people not in education, employment, or training (NEETs) has shown a remarkable increase in many European countries, such as Italy. The wide diffusion of NEETs represents an alarming social issue, as being NEET predisposes young people to long-term unemployment and social exclusion. It also has a significant negative impact on the economic growth and welfare equilibrium of countries. The aim of this paper is to analyze the determinants of the NEET condition in Italy through a step by step procedure beginning with the identification of their main characteristics and then proceeding with a focus on specific homogeneous clusters of NEETs. The decomposition of the gaps in the probabilities of being NEET between the various clusters allows verifying how personal characteristics effectively act. Furthermore, the influence of unobserved factors in the professional condition of young people has been analysed in more detail through a bivariate selection probit model on the propensity to look for a job against the condition of being inactive. The results confirm the crucial role of the education system, as well as the importance of the economic and social disparities between gender and the Italian territorial districts.

## Introduction

In the last years, the share of young people identified with the acronym of NEET—not in education, in employment or training—increased in many European countries. This increase was particularly high in Italy, which was more severely hit by the global financial and economic crisis than other European countries. Looking at the 2005–2015 decade, at the EU-28 Member States level, the share of NEETs increased from 16.1% up to 17.2% in 2013 but returned to 16.1% in 2015. During the same period in Italy, the share of NEETs increased six percentage points, from 21.0 to 26.8%, the highest in Europe, surpassed by Greece by only 0.2%.

A long-term NEET status produces many serious consequences on the economy of a country. Even if calculating the economic cost of NEETs is a very complex exercise, the Eurofound researchers (Eurofound 2012a), through appropriate simulations, estimated a very high cost. In addition to the loss in terms of foregone earnings and potential economic output and productivity (SALTO-YOUTH Inclusion Resource Centre 2015), Eurofound included in their estimate the impact on public finances in terms of welfare schemes (such as unemployment benefits, child benefits, housing benefits, education-related allowances, among others), as well as additional health, welfare and criminal justice expenditure. Moreover, Eurofound’s study also takes into account missed gains for the economy in terms of lack of productivity. Besides the serious consequences on the economy, the NEET phenomenon represents an alarming social issue. Being NEET means remaining unproductive, losing the opportunity to improve human capital. It also produces the accumulation of several disadvantages that are usually predictors of future long-term unemployment and can lead to poor mental health, particularly depression, with further extra social costs for society (OECD 2014). The consequences are even more serious in cases of a longer period in NEET status. These include isolation, uncertain and low-wage employment, criminality, failing to build a family, higher risk of marital instability, etc. (Balan 2015).

The causes behind the NEET status are manifold and only partially originating from the economic downturn. This paper aims at investigating the determinants of the NEET status in Italy before and after the economic crisis, attempting to sketch the different profiles of young people sharing in this condition. Once identified the causes, it would be possible to offer suggestions to policy makers as to the most appropriate actions to put an end to this phenomenon. Due to the heterogeneity of young people included in the NEETs, in the analysis, it is useful to treat different clusters separately, identifying them according to some relevant aspects influencing their behaviour.

The paper is structured as follows: in the “Who are NEETs” section, the authors compare different definitions of NEETs and outline their peculiarities based on the most relevant literature. The “The statistical methodology” section outlines the methodology, while the “Descriptive findings” and “The determinants of NEET status” sections report the main findings. Finally, the “Conclusions” section offers a conclusion.

## Who are NEETs

In an era characterized by the aging of the European Union population, young people constitute a large economic and social potential; as through their economic activity, they create the basis for maintaining the European welfare state model (Rollnik-Sadowska 2016). Therefore, in recent years many researchers—economists, sociologists, and psychologists—have begun to study NEETs, their determinant factors, and the consequences of the NEET status among youths.

According to work status, the labour force framework classifies people as “employed”, “unemployed” and “inactive”.^1^ While “unemployed” includes persons actively looking for a job, “inactive” refers to those not searching and unemployed. Meanwhile, those who are “inactive” can be engaged in training or education. NEETs comprise “unemployed” and the “inactive” persons who are not in training or education.

Even if the massive rise of young people who are NEETs is a very recent phenomenon, the issue has been well known for many years. Indeed, the acronym NEET emerged for the first time in the UK in the late 1980s (Coles et al. 2002). Eurofound (2012b) defined NEETs as “persons typically aged between 15 and 24 years who, regardless of their educational level, are disengaged from both work and education and are therefore at a higher risk of labour market and social exclusion”. A similar definition is given by Eurostat, the International Labour Office (ILO) as well as other organizations, which calculate the NEET rate as the percentage of the population of a given age group that is not employed and not involved in further education or training.^2^ These organizations consider a wider age class, from 15 to 34 years. In this paper, the authors agree with this wider age class, as including people up to 34 years of age is more appropriate for taking into account the difficulties of transition from the educational system to work which, in countries with high youth unemployment rates, such as Italy, is a very long process, and young people tend to remain in the parental home for longer. Under this framework, the NEET category is meant to describe, regardless of the country’s educational and employment system structure, all young people who finished their studies, enter the labour market to search for a job or remain inactive (Assirelli 2013). Even if compulsory school refers only to children less than 16 years of age, the share of young people who finish their studies late is constantly on the rise. In recent years, the greater complexity and globalization of labour markets together with the job polarization increased the need to attain a high educational level. Also for young adults, there is often a trade-off between employment, which is an objective in the short-term, and major investments in education and in work experience (Caroleo and Pastore 2007).

The remarkable increase of NEETs during the economic crisis is certainly linked to the growth in unemployment rates. Indeed, on the one side, in times of crises, young people have been more penalized than older workers due to having less work experience in addition to weaker work contracts and frequent lower qualifications (Marelli and Signorelli 2015). Besides these, other factors referable to institutional determinants—taxes on labour, unemployment benefits, collective bargaining, minimum wages, labour market policies—which are usually oriented to protect other typologies of people from the crises, mainly older workers, have de facto contributed to the increase of the youth unemployment rate. Howeversss, on the other hand, the NEET status also depends on factors not related to youth unemployment, such as exclusion from education and training (Bell and Blanchflower 2011). In 2015, Italy showed the highest percentage of inactive young people, with a marked predominance of women (on the total inactive, 63% were women and the remaining 37% were men). They constitute more than half of the total NEETs and represent an alarming social phenomenon, as they do not take any action, remaining outside the labour force. Inactive NEETs were mainly discouraged potential workers (11% of females and 13% of males in 2005; 13% of females and 15% of males in 2015), young people with health problems or those who have to assist a relative. It is reasonable to conjecture that some of those who declare to be inactive are engaged in the underground economy. Another reason is connected to the condition of being a housewife. Among females, those who declare to be housewives made up 44% of NEETs in 2005 and 32% in 2015.

According to Eurostat, *discouragement* is a concept used to describe those who are without work and available to work but who are not searching for a job because they believe that no work is available. This status is, therefore, affected by the economic cycle. Other reasons connected to discouragement are a lack of knowledge about how or where to seek work, the inability to find a work matching their skill levels or the unavailability to move away when there are no jobs in their area of residence (International Labour Office 2015).

The housewife definition derives from a gender-stereotyped division of roles within the couple. It is related to the old male breadwinner model that assumes that women have to provide with home and family care. It is a social more than economic issue that the European Commission has been fighting for years, as it contrasts with the Europe 2020 Strategy goal connected with the achievement of 75% overall employment rates for the 20–64 aged European population. Furthermore, the definition of housewife is based on a subjective declaration, and therefore, it is compatible with the condition of discouraged or other inactive individuals. The condition of NEET could also depend on an efficient welfare state system. The availability of specific services of assistance for *sick* and *disabled people* could increase the propensity to work of those who declare to be inactive because they are sick or have to assist a family member (Coppola and Di Laurea 2016). Furthermore, the actual share of inactive individuals on the total NEETs could be, on the one hand, underestimated, as people often self-define their status in surveys and may prefer to be viewed as “unemployed” rather than “inactive” for reasons of social acceptability (Robson 2008). However, on the other hand, it could be overestimated, as people who self-report as unemployed or as inactive might actually work and earn some money illegally. Boeri and Garibaldi (2002) estimated that in Italy, approximately 45% of those classified as unemployed and 10% of those classified as inactive are actually working irregularly. These percentages should be even higher for young people that have less contractual power on the labour market. Finally, some authors suggest to exclude from NEETs the so called false NEETs, i.e. those who are technically in a NEET situation, but are so voluntarily, as they do not view their own NEET status as a problem for themselves (SALTO-YOUTH Inclusion Resource Centre 2015; p. 25).

Therefore, being NEET has multiple and often intertwined causes. This status is also affected by *personal* and *family-related* socio-economic factors. With reference to the *personal factors*, Ryan (2001) analyzed the whole period between the end of compulsory schooling and the attainment of full-time, stable employment in some European countries, Japan and the USA. He found that socioeconomic disadvantage and a low educational attainment are the common key driving forces for youth inactivity and unemployment across countries. In a study conducted for Eurofound (2012a), Mascherini and Ledermaier found that the probability of being NEET is three times higher for young people with a low educational level, 40% higher for youth with some kind of disability and 70% higher for young people with an immigration background, while living in rural areas increases this probability up to 1.5 times (Eurofound 2012a; p. 3). On the same ground, Brunello and De Paola (2014) and Furlong (2006) highlighted that leaving school early and educational disaffection are often associated to the NEET status. “Early school leavers” are defined by Eurostat as “young people who leave education and training with only lower secondary education or less and who are no longer in education” (Eurostat 2016). In 2015, Italy showed the highest percentages of “early school leavers” across the EU (15%), just surpassed by Spain, Malta and Romania, where this phenomenon reached about the 20%. Restricting the analysis to NEETs, Italian early school leavers reach 39%, with a weak prevalence of women (52 vs. 48%). However, even among the highly educated, the mismatch between the skills acquired and those required by the labour market (Caroleo et al. 2018), in particular skill shortages in STEM (Science, Technology, Engineering, Mathematics) fields, makes young people more at risk of the NEET status (European Parliament 2015). Other personal characteristics predisposing to the NEET condition concern the status of young care leavers, substance abusers, young offenders, and for women, having a baby at an early age (Eurofound 2012a; p. 67). Moreover, a scarce trust in institutions, the lack of participation and interest in politics and scarce social participation are typical behaviours and attitudes connected with the NEET status (Alfieri et al. 2015). According to the personality traits predisposing to NEET status, an individual’s ability to persevere with long-term goals and the extent to which an individual believes that they can affect and control events play a significant role (Almlund et al. 2011; Mendolia and Walker 2015). Conversely, high cognitive abilities strongly protect from the risk of being NEET (Gladwell et al. 2016).

Finally, regarding *family-related factors*, a low social extraction and social exclusion are factors linked to the NEET status (Bynner and Parsons 2002; Robson 2008). They are also strictly associated with a low educational level (Thompson 2009). A low family income, a rented accommodation, low-skilled and low educational levels in parents are risk factors for NEET status (see among the others Scottish Executive 2005; Eurofound 2012a).

## The statistical methodology

The approach of analysis chosen by the authors follows the suggestions of Furlong (2006), who states that given the heterogeneity of NEETs, research and policy studies must begin by disaggregating demographic data. In this way, the distinct characteristics and needs of the various sub-groups can be identified.

Indeed, diverse policy implications correspond to different shares of the two components—unemployed and inactive non-student youths—and to the NEET group’s composition in terms of gender, area of residence and other important personal characteristics. Therefore, starting from a step-by-step guide, the objective consists in deciphering the meaning of the NEET rate to propose valid policy responses. Previous studies highlighted, for example, that when NEETs are more concentrated among the younger population (15–20 or 20–24 years), appropriate re-training programmes, apprenticeships and mentoring programmes encouraging more inclusive education for the less educated are more suited to face high unemployment rates. Otherwise, if the 25–30 years of age or older class are interested, policies oriented to fight structural unemployment should be more adequate (International Labour Office 2015). The following section considers other characteristics that have yet to be fully explored.

Indeed, the results of some first descriptive statistics (reported in Table 1, in the “The statistical methodology” section) suggest the estimation of a probit model evaluating the influence of personal and family characteristics on the probability that a young person is not NEET (EET,^3^ in employment, education or training). The statistically significant covariates *s* identify the determinants of NEET status. The model is as follows:

**Table 1:** Share of NEETs in the Italian macro-regions according to some personal characteristics^(*)^

	North-West	North-East	Centre	South	Isles	Italy
Year 2015						
Age class						
15–18	8.52	7.48	9.53	14.64	15.54	11.00
19–24	25.14	20.00	25.38	40.13	44.83	30.97
25–29	22.93	23.98	27.91	44.9	49.03	33.22
30–34	19.15	18.74	23.22	46.55	46.97	29.95
Education						
Low	20.10	17.5	20.47	37.36	41.65	27.72
Medium	20.17	18.26	23.57	37.81	39.54	27.41
High	13.95	16.88	20.27	35.27	35.62	22.49
Father’s education						
Low	19.56	17.6	24.02	39.89	43.77	30.49
Medium	13.59	11.96	17.96	24.04	24.29	18.01
High	10.19	8.58	13.36	17.11	17.1	13.05
Mother’s education						
Low	21.68	18.74	25.72	41.32	44.93	32.10
Medium	14.22	11.72	16.89	23.78	25.25	17.75
High	10.6	11.59	12.45	16.96	14.02	13.04
Civil status						
With partner	30.80	29.60	30.06	53.84	53.57	39.12
No partner	16.61	15.11	20.06	33.98	37.18	24.18
Nationality						
Immigrant	34.24	35.15	33.35	44.98	40.72	35.72
Native born	15.88	13.87	19.66	36.88	40.03	25.48
Field of study						
General	13.82	13.04	15.83	22.52	25.44	18.18
Humanities	20.21	22.37	25.85	42.88	44.7	29.75
Social	19.02	19.41	25.64	43.35	46.89	30.21
Science	16.64	13.62	20.49	37.66	39.38	23.66
Agrarian	13.27	15.59	17.43	37.55	34.51	21.63
Health	12.9	11.19	18.76	37.24	28.42	20.88
Services	27.91	23.42	32.71	52.98	54.13	38.34
Gender						
Men	15.74	12.59	19.21	33.45	37.53	23.20
Women	22.57	23.05	24.77	41.29	42.7	30.47
Year 2005						
Age class						
15–18	8.64	7.25	8.33	16.60	17.08	11.99
19–24	13.38	11.46	17.28	32.55	37.09	22.67
25–29	14.21	13.08	19.40	38.55	39.09	24.33
30–34	12.55	12.12	18.96	38.76	37.76	22.57
Education						
Low	15.52	13.80	17.54	36.17	37.08	25.12
Medium	10.05	8.90	15.28	27.54	29.26	17.34
High	12.40	14.02	20.32	34.11	29.41	20.45
Father’s education						
Low	10.54	8.92	15.27	30.74	32.06	20.64
Medium	8.06	6.54	10.27	17.18	15.08	11.37
High	6.15	7.68	10.44	11.81	10.28	9.47
Mother’s education						
Low	10.83	9.06	15.69	31.50	33.04	21.06
Medium	8.35	7.06	11.02	14.96	15.15	11.04
High	5.52	8.83	9.42	13.15	10.11	9.63
Civil status						
With partner	20.14	19.59	25.73	48.15	46.49	31.86
No partner	9.76	8.70	13.94	26.99	28.67	17.33
Nationality						
Immigrant	27.69	25.30	29.96	38.79	42.73	28.80
Native born	10.91	9.80	15.66	32.11	33.18	20.46
Field of study						
General	33.17	29.16	59.40	28.89	66.79	39.39
Humanities	13.23	11.84	17.94	28.10	28.14	20.05
Social	11.75	11.34	19.28	34.12	34.78	21.37
Science	6.81	6.59	11.20	20.79	22.46	12.51
Agrarian	10.05	10.94	11.99	33.34	39.81	19.37
Health	8.74	12.02	16.31	28.27	29.75	17.37
Services	15.72	15.63	22.02	39.98	45.87	26.32
Gender						
Men	7.57	6.32	11.79	23.61	25.17	14.59
Women	17.59	16.67	21.73	40.94	41.59	27.51

Total number of Italian young people analyzed is 12,774. Three thousand four hundred twenty-one of them are NEETs

Source: Ad hoc elaborations on Labour Force Survey data (2015–2005)

1 $$ {y}_i^{\ast }={X}_i\gamma +{v}_i\kern1em \mathrm{with}\kern1em {v}_i\sim N\left(0,{\sigma}_v^2\right) $$

where the latent variable ${y}_1^{\ast }$ drives the observed outcome of being EET (*y*_*i*_ = 1) through the following measurement equation:

2 $$ {y}_i=1\kern0.5em \mathrm{if}\kern0.5em {y}_i^{\ast }>0\kern1em \mathrm{and}\kern1em {y}_i=0\kern0.5em \mathrm{if}\kern0.5em {y}_i^{\ast}\le 0 $$

Subsequently, the authors provide estimations of different probit models for clusters of NEETs identified according to the modalities of some significant determinants. In this way, the different dynamics driving in these clusters versus the NEET status can be identified. The groups are the following:

-women versus men: in light of their different propensity to work. Indeed, the Italian female participation rate in the labour market is the lowest one when compared to the other European countries;
-residents in the Centre-North region versus residents in the South and Isles of Italy: in light of the high economic and social territorial disparities existing between these geographical areas (OECD 2016; p. 19; Eurostat 2015);
-young people with a scientific background of studies versus a non-scientific background (hard sciences vs. soft sciences): besides guaranteeing more employment opportunities, a scientific field of study usually represents a more demanding educational path, which is typical of young people that have a positive attitude towards education and the labour market or tend to accept only more qualified jobs;
-low and medium levels of education versus high levels: education is the most important human capital indicator, and a high educational level is the most important driver versus having a qualified job;
-young people with at least one highly educated parent versus others: to consider the influence of family background on students’ attitudes towards education and the labour market;
-young people looking for a job versus inactive youth.

In order to verify the determinants of the differences in the estimated probabilities of being not NEET between these clusters, an extension of the Oaxaca Blinder decomposition (Oaxaca 1973; Blinder 1973) was applied to these gaps. This decomposition is usually used for linear models in the fields of the classical Mincerian regression model, where it assumes the following form, according to the revisited and more generalized formulation of Oaxaca and Ramson (1994):

3 $$ {\overline{Y}}_A-{\overline{Y}}_B=\left({\overline{X}}_A-{\overline{X}}_B\right){\beta}^{\ast }+{\overline{X}}_A\left({\beta}_A-{\beta}^{\ast}\right)+{\overline{X}}_B\left({\beta}^{\ast }-{\beta}_B\right) $$

where the subscripts “A” and “B” identify, respectively, the most advantaged and the most disadvantaged group; *β*^∗^is a weighted average of the coefficient vectors *β*_*A*_ and *β*_*B*_:

4 $$ {\beta}^{\ast }=\Omega {\beta}_A+\left(1-\Omega \right){\beta}_B $$

Ω is a weighting matrix and *I* is the identity matrix. Following this approach, the Oaxaca-Blinder decomposition represents a special case of this generalized equation, in which Ω is a null matrix or the identity matrix, so that it assumes the following simplified form:

5 $$ {\overline{Y}}^A-{\overline{Y}}^B={\beta}^A\left({\overline{X}}^A-{\overline{X}}^B\right)+\left({\beta}^A-{\beta}^B\right){\overline{X}}^B $$

In the second member of both (3) and (5), the first term represents the difference in the mean characteristics of young people belonging to the two different groups (advantaged and disadvantaged group), valued at the return rate of the advantaged group (“endowment effect”, including, however, the pre-market discrimination). The remaining part at the second member shows the share of gap due to the different evaluation received by the same characteristics into the two models (“coefficients effect”), valued considering the differences in the regression coefficients estimated in the two models. Therefore, it represents the part of the gap connected with discrimination.

In the non-linear case, the generalized equation is:

6 $$ {\overline{Y}}_A-{\overline{Y}}_B=\left[{E}_{\beta \ast}\left({Y}_{iA}|{X}_{iA}\right)-{E}_{\beta \ast}\left({Y}_{iB}|{X}_{iB}\right)\right]+\left[{E}_{\beta_A}\left({Y}_{iA}|{X}_{iA}\right)-{E}_{\beta \ast}\left({Y}_{iA}|{X}_{iA}\right)\right]+\left[{E}_{\beta \ast}\left({Y}_{iB}|{X}_{iB}\right)-{E}_{\beta_B}\left({Y}_{iB}|{X}_{iB}\right)\right] $$

It decomposes the gap in the probability of being not NEET into the part due to the personal characteristics and into the part reflecting how these characteristics were rewarded. In this case, the advantaged and the disadvantaged groups are identified dividing young people according to the modalities of a dichotomous variable observed on them (males vs. females, north vs. south, etc.). The output of this decomposition shows the results for (1) Ω = 1, which uses as weights the coefficients of the advantaged group, (2) Ω = 0, which uses as weights the coefficients of the disadvantaged group, and (3) Ω strictly included between 0 and 1 and calculated following Newmark (1988). According to Newmark, the weight mechanism derives the “wage structure” from a theoretical model of discriminatory behaviour on the basis of the relationship between the form of employers’ discrimination tastes and the resulting estimate of wage discrimination. In this paper, the authors adopted this latter approach with the aim to distinguish how the different conditions of young people affect the probability of being EET, regardless by their personal characteristics. In other words, the authors aim at quantifying how the gap in the probability of being EET depends by the personal characteristics and how it depends on other factors which reflect on the values of the coefficients that differ into the two groups. This difference highlights a disparity that can be assimilated to discrimination.

Finally, in the analysis of the different propensity to look for a job among NEETs, it is important to consider that the job search is conditional upon active participation to the labour market (and the NEET status) and the current levels of young NEETs may discourage the personal propensity of actively looking for a job. Therefore, in order to verify and correct for the potential bias arising from the fact that the decision to engage in job search is just observed when a young person is not employed and not engaged in training, the authors estimate a bivariate probit model including the two-stage Heckman procedure (Green 1997). It allows addressing for the potential overlap in unobserved characteristics influencing both the young’s propensity to be a NEET and the behaviour of actively looking for a job as well analysing the mechanisms underlying the selection effect across these two different groups of young people.

Thus, the first probit model expressed by (1) assesses the influence of personal and family characteristics on the probability of being an EET. Subsequently, focusing on the subset of young people not employed and not involved in training and education, the probability of being actively looking for a job can be estimated by:

7 $$ {S}_i^{\ast }={X}_i^K\gamma +{W}_i^K\delta +{\varepsilon}_i^K\kern1em \mathrm{with}\kern1em {\varepsilon}_i^K\sim N\left(0,{\sigma}_{\varepsilon}^2\right) $$

where *K* identifies those for which the decision of looking for a job is observed. It includes a set of additional covariates (*W*) concerning the personal educational level, assumed as the most important indicator of human capital and job search behaviour. The two models’ joint error structure is defined as:

8 $$ \left(\begin{array}{l}{\varepsilon}_{i1}\\ {}{\varepsilon}_{i1}\end{array}\right)\sim \mathrm{Normal}\left(\left(\begin{array}{l}0\\ {}0\end{array}\right),\left(\begin{array}{l}1\kern1em \rho \\ {}\rho \kern0.75em 1\end{array}\right)\right) $$

In this way, it is possible to consider the potential for unobserved heterogeneity that could produce a correlation between the error terms in the two probit models. Therefore, not only the true effects of searching a job but also the effect on professional condition of having these unobservable characteristics are captured (Fleming and Kler 2011). If the error terms *v*_*i*_ and *ε*_*i*_, jointly distributed as bivariate normal with zero means and unit variances, are significantly and positively correlated (*ρ* > 0), unobserved factors increase both the probability of being not engaged in work or education and of looking for a job; for significant negative *ρ*, the reverse is true. Finally, a not significant *ρ* shows the absence of selection effect and the equivalence of using the bivariate or two separate probit models.

Data are from the Labour Force Survey, currently the main European reference source for comparable and multidimensional socio-economic statistics on employees and working conditions. The 2005 and 2015 waves allow verifying also the magnitude of NEETs and their characteristics before and after that the global economic and financial crisis produced its effects.

## Descriptive findings

During the last decade, the recession exacerbated the economic disparities across European countries, with more pronounced increases in unemployment rates—especially among youths—and in the NEET rates in the Mediterranean countries, which were more severely hit (Salvà-Mut et al. 2017). For many countries, these disparities also increased within them at a regional level. Italy shows very different levels of economic development between the Centre-North and the South and Isles areas. These disparities also concern the indicators related to the youth professional condition and their human capital. In 2015, early school leavers in the Isles amounted to three times those of the North-East regions (more than 24% against percentages less than 9%, respectively). The share of young people aged 30–34 who attained at least a 3-ISCED level of education was higher than 80% in the North-East and in many regions of the Centre, while in Campania, Puglia and Isles, it was approximately 60%. The low percentages of higher education directly influence the number of people at high risk of unemployment. The unemployment rates for youth 15–24 years of age in the South and Isles range between 50 and 60%, more than double of those living in the North-East and in Emilia Romagna, where they do not reach 30% (Eurostat online database and ISTAT datawarehouse). Southern Italy shows the highest percentages of NEETs but also a major incidence of the phenomenon in older age groups, suggesting that in this area, the transition from the educational system to work is a very long period (Table 1). While in the South, 1 out of 2 young people are NEETs, in the Centre-North, only 1 out of 4 belongs to this cluster. However, the comparison between the share of NEETs in 2015 and in 2005 shows that the increase was greater for men (8.61%) than for women (2.96%) while according to the territorial districts, while for men the highest increases concerned the residents in the Southern of Italy, further increasing the territorial gap, for women the highest increases affected those residing in the North-Centre of Italy, while in the South this share remained almost stationary. Bruno et al. (2014), which analysed the impact of the crisis on the NEET rates, highlighted the persistence of NEET rates and found that the increase of NEETs during the crisis was close to the youth unemployment rate while the sensitivity of NEET rate to GDP decreased. Among immigrants, the share of NEETs also differs widely across the Italian macro-regions. In the North, the gap in the share of NEETs between immigrants and native-born is very high and has increased after the economic crisis. Conversely, in the South, the gap between immigrants and the local population is very small and in the Isles further decreased after the economic crisis.

Education plays a significant role as predictor of NEET status: the percentages of NEETs are lower in the subset of highly educated persons. However, in the North-West, the gap in the share of NEETs between highly educated youth and other youth was of 7% in 2015, but in the Centre and South, it consisted only of 2%. The level of education attained by parents also affects the share of NEETs: between young people with a highly educated parent, NEETs were only 13% against percentages higher than 30% in the subset of youth with a lowly educated parent. The NEET status is more widely diffused among females and among young people living with a partner. In any event, it is reasonable to expect that living with a partner increases the probability of being a NEET only for women, due to the prevailing male breadwinner model. Finally, with reference to the field of study, which is distinguished only for young people with a medium or a high level of education, fields related to the service industry show the highest percentages of NEETs everywhere. This field of study corresponds only to a medium educational level, while the lowest percentages of NEETs refer to young people choosing general programmes of studies, medicine and agricultural fields.

The comparison with the same data for 2005 highlights that in the last decade, the highest increases in the share of NEETs have concerned mainly young people 19 to 29 years of age with a medium and not specialised level of education and lowly educated parents (Table 1).

## The determinants of NEET status

The probability of being EET was calculated through a classical probit model, in which the dichotomous variable denoting the status of EET is regressed on a set of covariates presumably influencing it. These covariates were chosen considering the literature on NEETs and the descriptive statistics analysed in the previous section. Similar models were estimated on sub-groups identified according to relevant characteristics connected with EET status (area of residence, field of study, level of education, behaviour related to the job search, parents’ educational level, gender). In general, these models highlight that the probability of being EET decreases as age increases, confirming that the NEET phenomenon involves mainly the older age classes (Table 2). Instead, a higher educational level, also related to parents, increases the probability of being EET, and the coefficients significance for the high educational level increases going from the 2005 to 2015 estimates. With reference to the field of study, going from the general programmes to all the other fields of study decreases the probability of being EET. This fact could be related to the long and difficult process of transition from the educational system to the labour market (Table 2).

**Table 2:** Probit models on the probability of not being a NEET

NEETs	All	Area of residence		Field of study		Level of education		Behaviour		Parent’s education level		Gender	
		South-Isles	North-Centre	Soft sciences	Hard sciences	Not high educated	High educated	Not looking for a job	Looking for a job	No high educated parent	High educated parent	Women	Men
2015													
In education, employment or training													
Gender (male = 1)	.038	.047	.041	.029	.145^*^	− .007	.071	.119^***^	− .094	.000	− .122	–	–
Age	− .035^***^	− .057^***^	− .019^***^	− .047^***^	.042^***^	− .032^**^	.010	− .035^***^	− .027^**^	− .049^***^	− .062^*^	− .044^***^	− .029^***^
Educational level (ref. low)													
Medium	.239^***^	.458^***^	.060	.104^***^	− .253^***^	–	–	.294^***^	.584^***^	.227^***^	− .009	.246^***^	.249^***^
High	.567^***^	.838^***^	.332^***^	.269^***^	–	–	–	.590^***^	1.06^***^	.624^***^	.233	.602^***^	.556^***^
Parents’ educational level (high = 1)													
Father	.201^***^	.286^***^	.141^*^	.235^***^	.215	.430^***^	− .079	.148^**^	.117	–	–	.247^***^	.172^**^
Mother	.204^***^	.364^***^	.124^*^	.281^***^	− .035	.375^***^	− .072	.116^*^	.272	–	–	.247^***^	.172^**^
Civil status^(1)^	− .479^***^	− .450^***^	− .467^***^	− .479^***^	− .339^***^	− .619^***^	− .143	− .685^***^	− .385^*^	− .499^***^	− 1.171	− .402^***^	.589^***^
Native born^(2)^	.374^***^	− .054	.504^***^	.308^***^	.424^***^	.344^***^	.680^***^	.364^***^	.091	.310^***^	.431^**^	.504^***^	.210^***^
Area^(3)^	.622^***^	–	–	.597^***^	.727^***^	.617^***^	.643^***^	.663^***^	.268^***^	.562^***^	.219^**^	.630^***^	.613^***^
Field of study (ref. general programmes)													
Humanities	− .411^***^	− .540^***^	− .314^***^	–	–	− .332^***^	−.168	− .291^***^	− .405^*^	− .522^***^	− .785^***^	− .386^***^	− .527^***^
Social	− .285^***^	− .429^***^	− .171^***^	–	–	− .087^**^	−.117	− .217^***^	− .357^***^	− .426^***^	− .827^***^	− .249^***^	− .347^***^
Science	− .199^***^	− .364^***^	− .078	–	–	− .040	.089	− .132^**^	− .444^***^	− .291^***^	− .431^***^	− .252^***^	− .202^***^
Agrarian	− .080	− .200	− .019	–	–	.104	.070	− .071	− .245	− .186	− .351	− .046	− .110
Health	− .121	− .333^**^	.038	–	–	− .291	.129	.035	− .378	− .483^***^	− .565^**^	− .067	− .310^*^
Services	− .501^***^	− .672^***^	− .371^***^	–	–	− .341^***^	− .201	− .392^***^	− .606^***^	− .613^***^	− 1.013^***^	− .527^***^	− .474^***^
male*civil status	1.154^***^	1.179^***^	1.177^***^	1.148^***^	.941^***^	1.258^***^	.857^***^	1.417^***^	.247	1.026^***^	2.436^*^	–	–
Intercept	.751^***^	1.610^***^	.925^***^	1.071^***^	− 1.187^***^	.803^***^	− .529	.965^***^	− 1.072^***^	1.177^***^	2.317^***^	.804^***^	.783^***^
Prob > chi2	0.000	0.000	0.000	0.000	0.000	0.000	0.000	0.000	0.000	0.000	0.000	0.000	0.000^***^
Pseudo R2.	.103	.070^***^	.070	.108	.100	.112	.072	.140	.068	.077	.140	.128^***^	.069^***^
*N*	106,917	40,706	66,211	88,887	18,030	91,964	14,953	94,646	12,271	67,321	12,530	53,230	53,687
2005													
In education, employment or training													
Gender (male = 1)	.116^***^	.136^***^	.103^**^	.141^***^	− .043	.105^***^	.101	.166^***^	− .099	.060^*^	.012	–	–
Age	− .021^***^	− .036^***^	− .007^*^	− .027^***^	.024^***^	− .019^***^	.033^***^	− .020^***^	− .029^**^	− .027^***^	− .034^**^	− .029^***^	− .012^***^
Educational level (ref. low)													
Medium	.202^***^	.176^*^	.178^**^	.263^***^	.743^***^	–	–	.230^***^	.291	.088	− .238	.289^***^	.120
High	.044	.096	− .024	.086^*^	.585^***^	–	–	.131	.433^*^	− .139	.795	.173^*^	− .099
Parents’ educational level (high = 1)													
Father	.234^***^	.428^***^	.073	.249^***^	.261^*^	.455^***^	− .043	.145^*^	.242	–	–	.239^**^	.256^**^
Mother	.208^***^	.314^***^	.106	.227^***^	.200	.403^***^	−.086	.166^*^	.162	–	–	.254^**^	.184^*^
Civil status^(1)^	− .673^***^	− .703^***^	− .645^***^	− .649^***^	− .741^***^	− .769^***^	− .145	− .874^***^	− .471^**^	− .726^***^	− .412	− .604^***^	.650^***^
Native born^(2)^	.357^***^	− .057	.449^***^	.364^***^	.444^***^	.341^***^	.446^***^	.369^***^	− .100	.176	− .255	.501^***^	.108
Area^(3)^	.738^***^	–	–	.755^***^	.661^***^	.764^***^	.594^***^	.739^***^	.125	.680^***^	.243^**^	.772^***^	.691^***^
Field of study (ref. general programmes)													
Humanities	.136^**^	.299^***^	.029	–	–	.318^***^	.497	.172^**^	.427^*^	.132	.198	.135^*^	.082
Social	.037	.075	.031	–	–	.251^**^	.465	.105	.215	− .021	− .163	.078	− .054
Science	.190^***^	.280^***^	.152^*^	–	–	.337^***^	.776	.213^***^	.359	.202^***^	.318	.284^***^	.146
Agrarian	− .079	− .155	.020	–	–	.076	.550	.007	.073	− .066	− .185	− .081	− .062
Health	.325	.407^**^	.287^*^	–	–	.165	.941	.359^***^	.275	.177	.170	.349^***^	.194
Services	− .211^***^	− .203	− .197^*^	–	–	− .071	.527	− .092^***^	− .084	− .280^***^	− .378	− .156	− .292^**^
male*civil status	1.418^***^	1.485^***^	1.441^***^	1.397^***^	1.424^***^	1.482^***^	1.135^***^	1.658^***^	.203	1.057^***^	1.014		–
Intercept	.467^***^	1.218^***^	.807^***^	.606^***^	− .987^***^	.477^***^	− 1.566^*^	.676^***^	− .653^*^	.895^***^	2.495^***^	.454^***^	.700^***^
Prob > chi2	0.000	0.000	0.000	0.000	0.000	0.000	0.000	0.000	0.000	0.000	0.000	0.000	0.000
Pseudo R2	.143	.113	.094	.143	.107	.160	.082	.190	.073	.071	.131	.156	.082
*N*	159,818	71,482	88,336	123,491	36,327	144,280	15,538	146,901	12,917	103,122	12,447	79,204	80,614

Source: Ad hoc elaborations on Labour Force Survey data (2015–2005)

^(1)^Living with a partner = 1; ^(2)^ Native born = 1; ^(3)^ Living in the North-Centre of Italy = 1

^***^Significance at 1%; ^**^significance at 5%; ^*^significance at 10%

A positive relationship can also be highlighted between the probability of being EET and Italian citizenship, residing in the North-Centre of Italy and the male gender, even if the significance of the coefficients related to gender decreases overtime (from 2005 to 2015 models). Gender is strictly related to civil status. Indeed, the probit models estimated distinguishing men from women highlight that living with a partner significantly decreases the probability of being EET for women; the opposite happens for men, however. Similar mechanisms in the determination of the probability of being EET are associated to the other covariates in these models. In any event, with reference to the field of study, with the exception of science and services, the positive impact on the probability of being EET is higher for men than for women. This could be because women are on average more educated than men, resulting in the probability that in the 15–34 age class, they are still studying for higher degrees. Furthermore, the parents’ high educational level exerts a greater influence on the status of EETs for women than for men. According to area of residence, even if the mechanisms driving the probability of being EET seem identical in the North-Centre and in the South and Isles, some peculiarities can be highlighted. Personal and parental education increases the probability of being EET more for young people living in the South than for residents in the North-Centre, while being born in Italy strongly increases the probability of being EET only in the North-Centre. When the clusters are identified according to the field of study, it is possible to note that, for young people choosing a non-scientific field of study, a medium level of education significantly increases the probability of being EET; instead, for others, a medium level of education decreases this probability. The group of young people having a low or a medium level of education is more likely to be NEET as age increases, while having a highly educated parent significantly decreases this probability. Conversely, for highly educated young people, the probability of being EET significantly increases when they were born in Italy and, in particular, in the North or Centre.

Finally, considering the behaviour looking/not looking for a job,^4^ the probability of being EET increases for all highly educated youths living in the North-Centre of Italy. Instead, only among young people that are not searching for a job, the probability of being EET significantly increases for young people with highly educated parents, for those born in Italy and for men.

The existence of different dynamics driving the probability of being EET between clusters of young people identified according to gender, area of residence, field of study, personal and parental level of education and propensity to look for a job suggests the need for an analysis of the gap in these estimated probabilities through decomposition techniques. In this way, it is possible to discover the gap determinants. In these decompositions, disadvantaged groups are identified as those with the lowest probability of being EET, i.e. “females”, “residents in the South of Italy”, “no scientific field of study”, “low personal educational level”, “parents’ low educational level” and “not looking for a job”. In the first rows, Table 3 shows the characteristic and component effects on the gap calculated using the coefficients of the advantaged group (omega = 1) as weights. In the groups based on gender and on area of residence, the personal characteristics highlight higher human capital features in the disadvantaged category. In other words, even if women and the residents in the South have higher human capital characteristics, they are more penalized for the performances reached, suggesting the presence of a possible penalty component.^5^ Indeed, the characteristics effect for 2015 is negative with percentages of 24 and 8%, respectively, on the total gap. Instead, the gap in the probability of being EET according to the level of education attained (high vs. low and medium) and field of study (hard sciences vs. soft sciences) has the same sign as the characteristics component, while the coefficient effect is negative. This result suggests that the advantaged groups have higher probabilities of being EET, even if the rewards favour the disadvantaged groups. Indeed, the characteristics effect has an incidence of 173% on the total gap.

**Table 3:** Oaxaca-Blinder decomposition of the probability of being not NEET between different groups of young people

Decomposit. components	Area of residence		Field of study		Education level		Look for a job vs inactive		Parents’ education level		Gender	
	Coef.	%	Coef.	%	Coef.	%	Coef.	%	Coef.	%	Coef.	%
2015												
Omega = 1												
Char	− .015^***^	− 8.106	.080^***^	173.31	.025	50.23	− .014^***^	1.948	.038^***^	29.162	− .017^***^	− 23.991
Coeff	.200^***^	108.110	− .034^***^	− 73.31	.025	49.77	− .703^***^	98.052	.092^***^	70.838	.090^***^	123.991
Omega = 0												
Char	.012^***^	6.545	.003	6.390	− .060^***^	− 119.53	− .029^***^	4.000	.058^***^	45.102	.009^***^	11.831
Coeff	.173^***^	93.450	.043^***^	93.610	.108^***^	219.53	− .689^***^	95.999	.071^***^	54.900	.064^***^	88.169
Omega = wgt												
Prod	.002^**^	1.219	.013^***^	28.126	− .006^*^	− 12.066	− .045^***^	6.274	.060^***^	46.062	.009^***^	12.077
Adv	.071^***^	38.224	.028^***^	59.570	.048^***^	95.414	− .593^***^	82.672	.059^***^	45.631	.031^***^	42.941
Disadv	.112^***^	60.56	.006^***^	12.300	.008^***^	16.650	− .079^***^	11.070	.011^***^	8.307	.033^***^	44.981
Raw	.185^***^	100	.046^***^	100	.050^***^	100	− .717^***^	100	.130^***^	100	.073^***^	100
*N*	7833+	4941	2254+	10,520	1951+	10,823	1510+	11,264	1368+	7597	6488+	6286
	12,774		12,774		12,774		12,774		8965		12,774	
2005												
Omega = 1												
Char	− .005^***^	− 2.582	.041^***^	40.473	.152^***^	2338	.001	− .118	− .009^***^	− 10.028	− .011^***^	− 8.860
Coeff	.196^***^	102.582	.060^***^	59.527	− .146^**^	− 2338	− .719^***^	100.118	.105^***^	110.028	.142^***^	108.86
Omega = 0												
Char	.001^***^	.683	.042^***^	42.322	.035^***^	539.15	− .032^***^	4.571	.018^***^	19.171	.028^***^	21.537
Coeff	.190^***^	99.317	.058^***^	57.678	− .028^***^	− 439.16	− .678^***^	95.429	.077^***^	80.829	.102^***^	78.463
Omega = wgt												
Prod	.005^***^	2.864	.045^***^	44.604	.031^***^	473.589	− .049^***^	6.845	.019^***^	20.112	.024^***^	18.515
Adv	.073^***^	38.266	.043^***^	42.842	− .022^***^	− 333.338	− .610^***^	85.731	.068^***^	71.122	.052^***^	39.949
Disadv	.112^***^	58.87	.013^***^	12.554	− .003^***^	− 40.251	− .053^***^	7.424	.008^***^	8.765	.054^***^	41.536
Raw	.191^***^	100	.101^***^	100	.006	100	−.711^***^	100	.095^***^	100	.130^***^	100
*N*	8748+ 5674		3285+ 11,137		1514+ 12,907		1150+ 13,272		1032+ 8367			

Source: Ad hoc elaborations on Labour Force Survey data (2015–2005)

^***^Significance at 1%; ^**^significance at 5%; ^*^significance at 10% obtained through bootstrap procedures

The weighting scheme according to Newmark (omega = wgt) considers the utility function capturing the discriminatory component (Newmark 1988).^5^ The productivity component highlights the gap due to a different productivity. It is near to zero for the groups based on the area of residence, gender and level of education, while it is even negative according to the behaviour looking/not looking for a job. Anyway, the most disadvantaged group consists in the residents in the South, where this component represents more than the 60% of the total probability gap, followed by gender, where it represents 45%. In the other cases, this component ranges from 8% (corresponding to the groups according to the parents’ educational level) to 16% (with reference to the personal educational level).

No particular differences arise from 2005 to 2015. However, the raw probability differential increases according to personal and parental educational levels, while for gender, it decreases.

Focusing on the analysis of determinants of behaviour unemployed versus inactive, for the purposes of limiting the risk of selection bias, a bivariate probit model allows estimating the probability of being actively searching for a job upon the unemployed condition (Castellano et al. 2013). Indeed, only 42% of NEETs are looking for a job, i.e. declared to be unemployed. Among those looking for a job, NEETs represent 90% of young people. The remaining 10% of people are engaged mostly in training or education. In the estimation of the model, several explanatory variables are tested according to a stepwise procedure. A first set of covariates detects some personal socio-demographic characteristics (i.e. gender, age, marital status, country of birth, macro-area of residence and the field of study), while a second set also includes information about personal educational level to explore the role of human capital characteristics in young peoples’ job search propensity (Table 4).

**Table 4:** Bivariate probit model for the probability of being not NEET and looking for a job

Actively searching for a job	All	Area of residence		Field of study		Parents’ educational level		Gender	
		South	North	Soft	Hard	No high	High	Women	Men
2015									
Male	.074^***^	.085^***^	.075^***^	.100^***^	− .023	.067^***^	.175^***^	–	–
Age	.022^***^	.036^***^	.009^***^	.030^***^	− .040^***^	.034^***^	.057^***^	.021^***^	.021^***^
Medium ed.	− .034^*^	− .157^***^	.153^***^	.120^***^	− .033^***^	− .022^**^	.308^***^	.151^***^	− .130^***^
High ed.	− .229^***^	−.328^***^	.100	.151^***^	–	− .305^***^	.277^***^	.099	− .401^***^
Father’s high ed.	− .193^***^	− .216^***^	− .210^***^	− .256^***^	− .190^***^	–	–	− .220^***^	− .176^***^
Mother’s high ed.	− .228^***^	− .354^***^	− .189^***^	− .312^***^	− .076	–	–	− .325^***^	− .159^***^
Civil status	− .317^***^	− .470^***^	− .228^***^	− .336^***^	− .208^***^	− .109	.898^**^	− .322^***^	− .416^***^
Native born	− .242^***^	.235^***^	− .359^***^	− .175^***^	− .357^***^	− .281^***^	− .547^***^	− .283^***^	− .204^***^
North-Centre	− .313^***^	–	–	− .274^***^	− .504^***^	− .292^***^	− .122^***^	− .273^***^	− .354^***^
Field of study									
Humanities	.442^***^	.533^***^	.218^*^	–	–	.484^***^	.583^***^	.237^**^	.559^***^
Social	.276^***^	.317^***^	.147^***^	–	–	.344^***^	.467^***^	.180^***^	.306^***^
Science	.188^***^	.326^***^	.007	–	–	.236^***^	.305^***^	.189^***^	.208^***^
Agrarian	.071	.222^***^	− .112	–	–	.120^**^	.373^**^	.056	.093
Health	.306^***^	.460^***^	.017	–	–	.485^***^	.279^**^	.121	.393^***^
Services	.430^***^	.496^***^	.326^***^	–	–	.491^***^	.696^***^	.401^***^	.413^***^
Male*civil status	− .113^***^	.039	− .232^***^	− .062	− .223^***^	− .379^***^	− 1.209^**^	–	–
Constant	− 1.392^***^	− 2.145^***^	− 1.369^***^	− 1.676^***^	.667^***^	− 1.623^***^	− 2.814^***^	− 1.458^***^	− 1.255^***^
Not NEET									
Male	− .075^***^	− .130^***^	− .101^*^	− .112^***^	− .021	− .084^***^	− .210^***^	–	–
Age	− .0009	.010^**^	− .015^***^	− .035^***^	.038^***^	.012^***^	− .062^***^	− .025^***^	− .001
Father’s high ed.	.022	.020	.250^***^	.283^***^	.217^***^	–	–	.292^***^	.024
Mother’s high ed.	.082	.138^*^	.256^**^	.349^***^	.160^***^	–	–	.447^***^	.123
Civil status	− .491^***^	− .602^***^	.063	.237^***^	.138^**^	− .361	− 2.720^*^	− .038	− .387^***^
Native born	− .075^*^	.311^**^	.353^***^	.169^***^	.391^***^	− .154^***^	.549^***^	.258^***^	− .046
North-Centre	.007	–	–	.321^***^	.521^***^	.056^*^	.102^*^	.335^***^	.028
Field of study									
Humanities	.317^***^	.393^***^	− .171	–	–	.377^***^	− .677^***^	–(*)	.372^***^
Social	.180^***^	.133^***^	− .140	–	–	.237^***^	− .630^**^	− .105	.158^***^
Science	.072^*^	.161^***^	− .095^**^	–	–	.069	− .355^**^	−.062	.008
Agrarian	.109	.031	.143	–	–	.219^*^	− .633^**^	− .149	.152
Health	.280^***^	.320^***^	.081	–	–	.286^***^	− .361^***^	.095	.273^*^
Services	.044	− .078	− .427^***^	–	–	.100^*^	− 1.210^***^	− .521^***^	.026
Male*staciv	.076	.135	.294^***^	.112^**^	.286^***^	− .345	1.167^**^	–	–
Constant	− 2.199^***^	− 2.771^***^	1.315^**^	1.709^***^	− .536^***^	− 2.400^***^	2.836^***^	.828	− 2.246^***^
Rho	.88^***^	.99^***^	− .95	− .99^***^	− .99	.91^***^	−.97^***^	− .81	.90^***^
Prob > chi2	0.000	0.000	.0000	0.000	0.000	0.000	0.000	.1225	0.000
Censored obs.	94,646	34,644	60,002	88,887	18,030	58,529	11,708	47,516	47,130
Uncensored obs.	12,271	6062	6209	78,990	15,656,	8792	822	5714	6557
*N*	106,917	40,706	66,211	9897	2374	67,321	12,530	53,230	53,687
AIC	9954		5.438		1.844		737		5261
2005									
Male	− .012	.023	− .056^***^	− .018	.082^***^	.003	− .074	–	–
Age	.010^***^	.024^***^	− .002	.015^***^	− .018^***^	.016^***^	.031^***^	.012^***^	.009^***^
Medium ed.	− .027	.039	− .078	.122^***^	− .630^***^	.056	.501^**^	− .007	− .046
High ed.	.224^***^	.339^***^	.145^**^	.285^***^	− .326^***^	.310^***^	1.012^***^	.245^***^	.196^***^
Father’s high ed.	− .272^***^	− .411^***^	− .161^***^	− .282^***^	− .244^***^	–	–	− .246^***^	− .285^***^
Mother’s high ed.	− .196^***^	− .312^***^	− .109^**^	− .212^***^	− .163^***^	–	–	− .183^***^	− .203^***^
Civil status	− .260^***^	− .405^***^	− .137^***^	− .315^***^	.010	− .093	− .128	− .275^***^	− .500^***^
Native born	− .246^***^	.128^*^	− .300^***^	− .193^***^	− .493^***^	− .212^***^	.087	− .309^***^	− .152^***^
North-Centre	− .483^***^	–	–	− .464^***^	− .558^***^	− .481^***^	− .214	− .420^***^	− .552^***^
Field of study									
Humanities	.062^*^	− .025	.129^**^	–	–	.024	− .187	.071	.053
Social	.130^***^	.066	.170^***^	–	–	.116^***^	− .037	.167^***^	.067
Science	− .046	− .104^**^	− .000	–	–	− .100^***^	− .275	− .038	− .046
Agrarian	.201^***^	.196^***^	.199^**^	–	–	.134^**^	.360	.299^***^	.152^**^
Health	− .095^*^	− .103	− .108	–	–	− .057	− .429	− .074	− .167
Services	.318^***^	.254^***^	.359^***^	–	–	.336^***^	.282	.361^***^	.254^***^
Male*civil status	− .249^***^	− .102^***^	− .460^***^	− .160^***^	− .664^***^	− .186	− 4.183^***^		
Constant	− 1.132^***^	− 1.848^***^	− 1.227^***^	− 1.313^***^	.338^***^	− 1.321^***^	− 2.733^***^	− 1.186^***^	− 1.135^***^
Not NEET									
male	− .083^**^	− .023	− .179^**^	− .032	− .133^*^	− .083^**^	− .137		
Age	− .029^***^	− .035^***^	− .026^***^	− .025^***^	− .002	− .032^***^	− .031	− .029^***^	− .031^***^
Father’s high ed.	.338^***^	.552^***^	.140	.295^***^	.359^**^	–	–	.343^***^	.306^***^
Mother’s high ed.	.237^***^	.294^***^	.210	.266^***^	.086	–	–	.247^**^	.215^*^
Civil status	− .286^**^	− .092	− .505^***^	.141^***^	− .498^***^	− .118	5.406^***^	− .273^*^	− .029
Native born	.030	.110	− .019	.155^***^	− .055	− .074	.648^***^	.085	− .036
North-Centre	.339^***^	–	–	.456^***^	.010	.365^***^	− .152	.364^***^	.296^*^
Field of study									
Humanities	.570^***^	.589^***^	.573^***^	–	–	.498^***^	.648^***^	.466^***^	.828^***^
Social	.355^***^	.343^***^	.383^***^	–	–	.226^***^	.201	.289^**^	.443^***^
Science	.555^***^	.528^***^	.592^***^	–	–	.410^***^	.517^*^	.552^***^	.589^***^
Agrarian	.195	.098	.305	–	–	.114	.558	− .052	.330^*^
Health	.500^***^	.479^***^	.512^**^	–	–	.087	− .099	.332^**^	.922^***^
Services	− .004	− .042	.065	–	–	− .078	− .507	− .017	− .027
Male*staciv	.283^*^	.018	.637^**^	.188^***^	.270	.233			
Constant	.326	.493	.265	1.480^***^	− 1.147^***^	.818^**^	− 1.525	.351	.193
Rho	− .539^***^	− .603^***^	− .369	− .970^***^	.290	− .658^***^	.505	− .563	− .485
Prob > chi2	0.000	0.000	.4544	0.000	0.367	0.000	0.2126	.0627	.2228
Censored obs.	146,901	83,852	63,049	33,694	113,207	93,518	11,810	72,522	74,379
Uncensored obs.	12,917	4484	8433	2633	10,284	9604	637	6682	6235
*N*	159,818	88,336	71,482	36,327	123,491	103,122	12,447	79,204	80,614
AIC	8572	4015		1793		491		4158	

^(*)^The dummy for humanities studies was dropped in order to guarantee the concavity of the log pseudolikelihood

Source: Ad hoc elaborations on Labour Force Survey data (2015–2″5)

^***^Significance at 1%; ^**^significance at 5%; ^*^significance at 10%

In contrast to the pre-crisis situation, the potential for unobserved heterogeneity produces a positive correlation in the probit models estimated on all the young people, indicating that the unobserved factors increase both the probability of being not NEET—i.e. being in education, employment or training—and belonging to the group that is looking for a job. Probably, the increased share of discouraged young people after the crisis contributed to these results. In other words, in 2015, those who were actively looking for a job were the most active and resolute young people. This double active attitude expressed by a positive correlation coefficient is not confirmed on the cluster of young people with a non-scientific field of study or with highly educated parent. In addition, the correlation between the two error terms is not significant for the residents in the North-Centre of Italy and for those with a scientific education. Probably, the higher job opportunities for the North-Centre residents and for young people with a scientific background affect these results. Furthermore, young people with highly educated parents are usually less pressed to find a job because a higher education habitually corresponds to a high standard of living, allowing students to continue to study or wait for a more qualified job. In a gender perspective, being male increases the probability of looking for a job. The greater female propensity to study could motivate the significant and negative sign of the male coefficient in the model related to the probability of being not NEET. Instead, living in the South of Italy or having parents with a low or medium level of education requires a major achieving attitude to confront future prospects (Table 4).

## Conclusions

The high share of NEETs in Italy represents an alarming social and economic issue. Indeed, young people not engaged in work, education or training constitute an obstacle to economic growth, hampering productivity and competitiveness for the whole country, especially when this condition persists for a prolonged period. They are exposed to a high risk of poverty and social exclusion, as they cannot improve their skills and competences, losing competitiveness. Furthermore, in an era of aging population, they constitute an obstacle to reaching the welfare state equilibrium. Conversely, the engagement of more young people in economic activities promotes an increase of productivity and social well-being (Eurostat 2016).

In this paper, the authors analysed the characteristics of NEETs with the aim of identifying the factors that majorly expose young people to this condition. In addition, they compare the NEETs’ conditions and characteristics before and after the economic crisis. The results in some cases contrast with the expectation of direct correlation between level of education and professional condition. Indeed, regardless of level of education and field of study, the share of NEETs is higher for women, residents of the South and Isles and immigrants. These groups suffer a disadvantage linked to many factors. They attain to the most adverse economic conditions of Southern of Italy, where youth unemployment rates are very high and to the stereotypes linked to gender and race. Women and immigrants are indeed still targets of direct or indirect forms of discrimination. Consequently, women and young people living in Southern of Italy, even when more educated than other young people, suffer a greater penalty. However, other external factors significantly affect these results. Some of them can be found in the lack of adequate services for persons of older age and child care—penalizing women above all, who assume the main role of care givers—the informal or underground economy, which engages irregular workers, especially among immigrants and residents of the South and Isles.

The economic crisis still worsened young people’s condition, increasing social inequalities. The comparison between the pre- and post-crisis conditions highlights that the share of NEETs increased mainly among the lowly educated young people and those with lowly educated parents. According to gender, men resulted more penalized while with reference to the territorial districts, young people living in the South suffered the higher increases. Further, after the crisis, the probability of moving from a temporary to an indefinite work status significantly decreased, while the rate of moving from temporary work to unemployment doubled, and the probability of becoming inactive became similarly greater (Dota 2011).

In the analysis of actions to be completed with the aim of decreasing the share of NEETs, it becomes necessary to distinguish between unemployed and inactive. When NEETs are looking for a job, the focus has to be directed to the labour market and the educational process; conversely, for the inactive, in addition to the educational process, other social factors assume a crucial role. Past Italian labour market reforms, carried out to reduce high unemployment rates, have been oriented to reduce fixed-term contracts and to favour flexibility. As a consequence, they have only reduced the actual levels of workers protections, in particular against dismissals, without creating concrete new job opportunities for young unemployed or inactive people (Directorate General for Internal Policies 2014). The recent reforms promoted by the EU, such as the youth guarantee schemes, consisting in apprenticeships and traineeships programmes or support schemes for young business starters, have not yet reached adequate results (European Commission 2017). Indeed, since May 2014, when the Youth Guarantee initiative was implemented, these programmes have been converted in concrete job opportunities in only a few cases. Actions performed to reduce the share of NEETs should be devoted to subsidising youths to remain in the educational system, due to the positive effect of a high educational level on employment (Maguire and Rennison 2005). Also, the promotion of measures that productively engage people should be encouraged. The reference is, for example, to vocational education and training programmes and opportunities for non-formal education and training, carried out to reduce the gap between school programmes and what the labour market requires. Therefore, it is fundamental to reform the education system and the school-to-work transition processes. Furthermore, adequate income support schemes and fiscal incentives could help orient young people in their choices (Caroleo and Pastore 2007).

With reference to inactive youth, in addition to decreasing irregular work, actions should be carried out to valorise women’s economic role, diminishing the male breadwinner model and increasing the welfare services for child and elderly care, whose absence constitutes an obstacle to women’s propensity to work. Furthermore, actions carried out to valorise the role of immigrants in the economy and society can be implemented. They could increase the competitiveness in the global economy in a framework of constantly changing demands of economies.

## Footnotes

1 The international standards and guidelines for measures of the economically active population are set out in the International Labour Organization Convention 160 and in the International Conference of Labour Statisticians Resolution 170 of 1982. Also, Eurostat adopted these guidelines in the Labour Force Survey.

2 Many studies proceed to calculate the ratio of NEETs adding to the inactive to the unemployed, including also students. However, ILO calculates the ratio having as numerator the difference between the number of youth and the sum of those in employment or in education or training. As the denominator, there is the total number of youth (International Labour Organization 2013).

3 To facilitate the results interpretation, 1 identifies young people not NEETs and 0 otherwise. In this way, positive coefficients for probit model covariates indicate a positive effect. This code is also due in the next step of the probability gap decomposition, requiring that the lowest probabilities have to be associated to the disadvantaged group, that is, NEET young people (Sinning et al. 2008).

4 Of course, within EETs, young people looking for a job are those who already work and search for other jobs or students that undertake job search actions. Instead, among the NEETs, youths looking for a job are unemployed.

5 In the literature of decomposition, it is common to refer to discrimination to indicate the discriminant behaviour of employers, which penalize in remuneration a particular group, such as blacks rather than whites or women, rather than men. In this case, it is more correct to refer to a disadvantage arising from particular characteristics, such as being female, living in the South and so on.
